# Cryopreservation of single-sperm: where are we today?

**DOI:** 10.1186/s12958-020-00607-x

**Published:** 2020-05-12

**Authors:** Shasha Liu, Fuping Li

**Affiliations:** 1 Human Sperm Bank, West China Second University Hospital, Sichuan University, Chengdu, 610041 China; 2 Key Laboratory of Birth Defects and Related Disease of Women and Children (Sichuan University), Ministry of Education, Chengdu, 610041 China

**Keywords:** Cryopreservation, Single sperm, Frozen carrier, Freezing method, Clinical application

## Abstract

**Background:**

Patients with severe oligospermia and nonobstructive azoospermia have very limited numbers of viable sperm in their epididymal and testicular samples. Thus, cryopreservation of their sperm is performed to avoid repeated sperm retrievals and to preserve their sperm from any side effects of any treatment regimens.

**Main body:**

The development of intracytoplasmic sperm injection technology has extended the therapeutic capacity of assisted reproductive technology for men with azoospermia via the surgical or percutaneous isolation of sperm from the testis/epididymis. The conventional cryopreservation techniques are inadequate for preserving individually selected sperm. The technique for freezing single sperm was first developed in 1997 and has been explored from the perspective of frozen carriers, freezing programs, and cryoprotectant formulations. Among these methods, advances in frozen carriers have directly improved single-sperm freezing technology. In this review, we evaluate the different technologies for the cryopreservation of single sperm by discussing the advantages and disadvantages of different freezing methods, their clinical applications, and the outcomes for a range of frozen carriers.

**Conclusion:**

Our review article describes the latest and current technologies implemented for the cryopreservation of single sperm that could potentially benefit patients with severe oligospermia and who rarely have any sperm in their ejaculate. This review provides a platform to understand the process and pitfalls of single-sperm cryopreservation to ensure further improvements in the cryopreservation technology in future studies.

## Background

In recent years, increasing environmental pollution owing to continuous industrialization and psychological stresses [1, 2] have resulted in an increase in infertility issues worldwide. Infertility in men now accounts for approximately 50% of the fertility-related problems [3]. Infertility is frequently observed in patients with cancer and in men with severe oligozoospermia or azoospermia owing to spermatogenic dysfunction. The primary challenge in clinical practice is ensuring that the patient is restored to good health after receiving the relevant treatments. However, specific treatment regimens (chemotherapy, radiotherapy, surgery) lead to adverse side effects such as impaired spermatogenesis, testosterone deficiency, or sexual dysfunction, thereby affecting the reproductive and psychological well-being of the patient [4, 5]. Most patients are concerned about the fertility-related side effects, regardless of the treatments received. Therefore, preserving fertility before treatment is critical to ensure the quality of life. In addition, etiological factors involved in male infertility factors also include congenital factors (anorchia, cryptorchidism, congenital absence of vas deferens, genetic abnormalities), acquired factors (testicular torsion, testis trauma, surgery etc.) and idiopathic forms. The etiology is still unknown in about half of the cases and it is termed “idiopathic infertility” [6]. The complete diagnostic workup is important for patients. In general, cryopreservation of sperm is a way to avoid risks.

Sperm banking was initially used for the cryopreservation of sperm [7] and was first developed to facilitate pregnancy in the 1950s [8]. Cryopreservation technologies are integral for managing male-factor infertility. Sperm donors are properly screened and quarantined before semen cryopreservation to eliminate the risk of transmission of HIV, hepatitis B and C, syphilis, and bacterial infections to the recipient. Advancements in intracytoplasmic sperm injection (ICSI) technology provide the opportunity to overcome fertility issues in patients with azoospermia (when the semen is devoid of sperm) and severe oligozoospermia. The ability to cryopreserve small numbers of spermatozoa in males afflicted with nonobstructive azoospermia using testicular sperm extraction (TESE) avoids the requirement for repeated surgery and promotes the preservation of fertility [9–13]. Conventional sperm preservation techniques can result in sperm loss owing to sperm adherence to the carrier vessel, harsh centrifugation, and washing procedures [14]. Thus, the conventional technique is particularly problematic in cases in which sperm numbers are low [15].

In 1997, Cohen et al. were the first to describe a novel cryopreservation technique for individual sperms using an empty zona pellucida (ZP) [16], thereby providing a theoretical and technical foundation for subsequent techniques. They introduced the concept of single-sperm freezing. Since then, a multitude of cryopreservation techniques have been developed to improve sperm counts. In this article, we review the single-sperm cryopreservation methods practiced in the last 20 years (Table 1), including the types of frozen carriers (Table 2), spermatozoa from different sources, prefreezing treatments, freezing procedures, resuscitation after freezing, and clinical applications. We performed an in-depth analysis of the advantages and disadvantages of these techniques to provide the theoretical basis for improving the single-sperm freezing technology in the future.

**Table 1:** Single-sperm cryopreservation methods

Carrier	Year	Sperm origin	Recovery rate	Motility rate	Survival rate	Fertilization rate
Human, mouse and hamster zonae [16]	1997	Ejaculate	73%	82%	NA	50%
Human and hamster zonae [17]	1998	Ejaculate, testicular	≥75%	67–100%	NA	65%
Human zona [18]	1999	Ejaculate	92%	NA	84%	NA
Human zonae [19]	2000	Testicular	88%	27%	NA	23%
Human and mouse zonae [20]	2000	Ejaculate, epidydimal, testicular	82%	83%	NA	NA
Mouse zonae [21]	2000	Testicular	100%	58%	77%	NA
Human zona pellucida [22]	2001	Epidydimal	NA	NA	NA	57%
Human zona [23]	2003	Ejaculate	59%	73%	NA	NA
*Volvox globator* algae [24]	2004	Ejaculate	100%	≥63%	NA	NA
Alginic acid drops [25]	2006	Ejaculate	NA	20–30%	40–50%	NA
Agarose gel microspheres [26]	2007	Ejaculate	98%	78%	81%	NA
Hollow-core agarose capsules [27]	2015	Ejaculate	94%	84%	95%	NA
Hollow hyaluronan-phenolic hydroxyl microcapsules [28]	2016	Ejaculate	95%	14%	NA	NA
Cryoloops [29]	2003	Ejaculate	NA	45%	NA	NA
Cryoloops [30]	2004	Ejaculate	68%	73%	NA	67%
Cryoloop [31]	2004	Epidydimal, testicular	72%	NA	NA	58%
Droplets on plastic dish [32]	2000	NA	90–100%	NA	NA	NA
0.5ul microdrop in a plastic dish [33]	2003	Ejaculate	100%	< 50%	NA	NA
5ul microdrop in a plastic dish [34]	2008	Testicular	100%	2%	NA	18%
Cell Sleeper [35]	2012	Testicular	83%	NA	NA	83%
Cell Sleeper [36]	2012	Ejaculate	100%	28%	58%	NA
Cell Sleeper [37]	2016	Testicular	94%	56%	NA	66%
Cryotop [35]	2012	Ejaculate, testicular	96%	NA	NA	64%
Cryotop [36]	2012	Ejaculate	100%	44%	78%	NA
Cryotop [38]	2011	Ejaculate, testicular	93%	43%	NA	NA
Cryoleaf [39]	2011	Ejaculate, epididymal	99%	66%	NA	61%
Cryopiece [40]	2017	Ejaculate, testicular	83%	48%	NA	73%
Closed slice [41]	2015	Ejaculate	94%	41%	70%	NA
Closed slice [42]	2017	Testicular	92%	17%	66%	56%
The novel sperm vitrification device [43]	2018	Ejaculate	96%	33%	NA	59%

**Table 2:** Summary of the different type carriers for single-sperm cryopreservation

Biological	Non-biological
Zona pellucida of different species	Polymerized alginic acid capsules
Volvox globator algae	Hollow-core agarose capsules
	Hollow hyaluronan-phenolic hydroxyl microcapsules
	Cryoloops
	Culture dish
	Cell Sleeper
	Cryotop
	Cryoleaf
	Cryopiece
	Closed slice
	The novel sperm vitrification device

### Single-sperm cryopreservation within the empty ZP

**Fig. 1:**
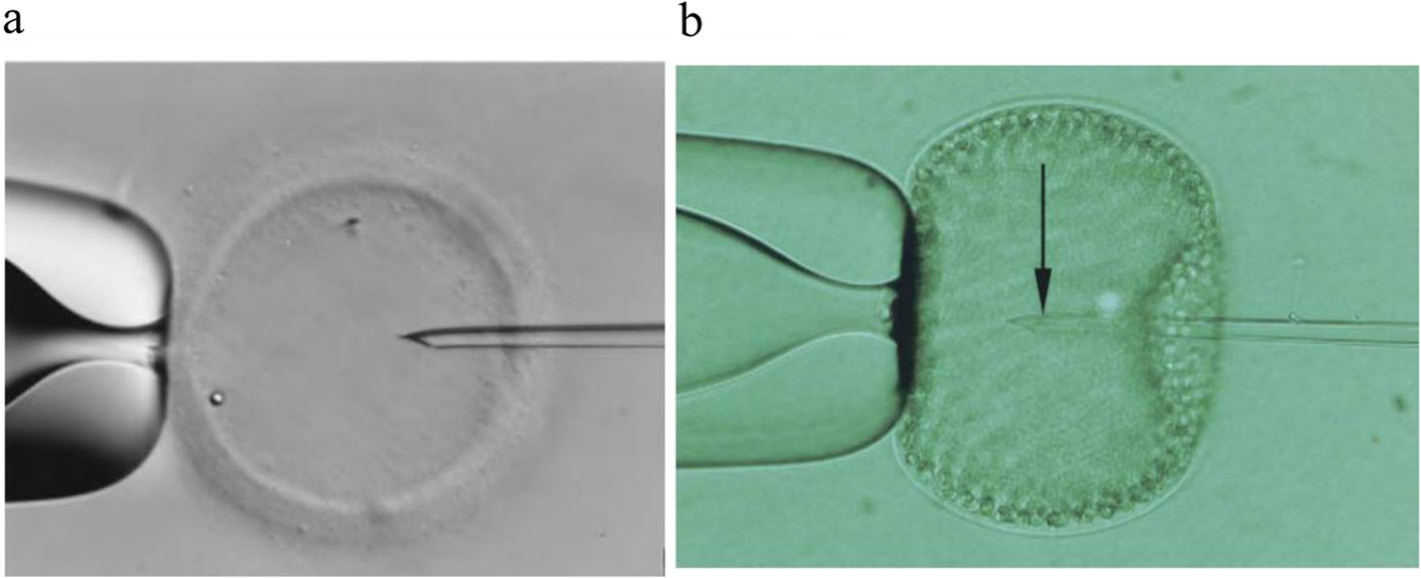
(**a**) (Adapted from Cohen et al. [16]) Empty pre-fertilization human zona. The needle was inserted into the zona. (**b**) (Adapted from Just et al. [24]) Volvox globator sphere, fixed to the holding pipette

Repeated TESE is both costly and invasive, and it can frequently have adverse effects on the testis, including deterioration of spermatogenesis, inflammation, irreversible atrophy, and partial testicular devascularization [12]. Repetition of these procedures can be avoided through the cryopreservation of spermatozoa. Although studies [44, 45] have reported sperm survival and the births of live offspring following the cryopreservation of epididymal and testicular sperm, conventional sperm freezing techniques result in low viability [46]. Therefore, it is crucial to develop new sperm freezing methods. The loss of spermatozoa through conventional addition and removal of cryoprotectants in relatively large volumes of media can be circumvented by the insertion of the spermatozoon into an enclosed porous capsule that can be correctly visualized and handled microscopically prior to and after cryopreservation. The chosen vehicle for such a purpose is the ZP that can be used following the removal of its cellular material. The cytoplasmic components in the oocyte ZP were removed, and individual sperm was injected into the empty ZP using an ICSI needle and loaded onto a frozen straw for cryopreservation (Fig. 1a). After thawing, the recovery and fertilization rates were 73% and 50%, respectively. Spermatozoa recovered from human zonae fertilized the same proportion of oocytes as fresh fertile control spermatozoa. The fertilization rate in human zonae was marginally lower (*P* < 0.05) than that for spermatozoa frozen in animal zonae [16]. Over the next 6 years, frozen vectors from the empty ZP of human, mice, hamsters, and golden hamsters were used [16–23, 47, 48]. There was no statistical difference in freezing and thawing results of human and mouse ZP as carriers [20, 47]. In 1998, Walmsley et al. [17] reported the first cases of live births after ICSI procedures by using empty ZP frozen testicular sperm.

The empty ZP is larger than the sperm, and it forms a transparent matrix that is easy to observe and manipulate through microscopy. However, inevitable shortcomings limited their development and ZP was replaced later with non-biological carriers. The empty ZP of animals acts as a biological carrier, but it has limited availability and bioethical issues. New FDA and European Tissue Directive regulations regarding the exposure of human gametes and embryos to animal products make the use of rodent zonae less feasible. Furthermore, human zonae availability is also very restricted. To use an empty ZP as a frozen carrier, a hole is punched in the ZP using a laser. However, drilling small holes can increase the risk of leaving some host DNA fragments behind. The transfer of foreign DNA using sperm as vectors during ICSI has been reported previously [49, 50]. On the other hand, drilling larger holes can lead to the risk of sperm loss. Furthermore, attempts to seal the hole using oil droplets has limited effectiveness and interferes with sperm recovery post-thaw [18, 23]. Sperm and ZP3 binding on the human ZP also induces acrosome reactions, thereby frequently affecting sperm quality [16].

### Single-sperm cryopreservation within the *Volvox globator* sphere

The *Volvox globator* is an alternative in countries that prohibit the destructive use of oocytes, even after fertilization has failed. In 2004, Just et al. [24] used the *Volvox globator* as a single-sperm cryopreservation vehicle (Fig. 1b). Spherical colonies (diameter 0.5–1.0 mm) contain 1500–20,000 peripheral cells. Considering its size and green color, the *Volvox globator* is easy to identify and operate. Eight sperm were frozen in each algae ball, and three Volvox spheres were placed in a frozen straw at 4 °C for 10 min. The straws were frozen on liquid nitrogen (LN_2_) vapor for 10 min before submerging in LN_2_. The spermatozoa recovery rate was 100%, and motility rate was at least 60% after thawing. The use of the spherical algae *Volvox globator* offered a promising approach to the cryopreservation of functional motile sperm. A drawback of the Volvox sphere was the transfer of genetic material from the algae to the egg cells, which requires further investigation. Moreover, the new FDA and European Tissue Directive regulations consider the use of algae (non-human tissue) to store human sperm unacceptable in a clinical setting.

### Single-sperm cryopreservation within non-biological carriers such as empty ZP

Sperm injection into an empty ZP is easier for technicians because the procedure is similar to that of ICSI [15]. However, concerns remain regarding the use of ZP, including bacterial and virus contaminants from animals. To overcome these challenges, hollow capsules similar to empty ZP have been fabricated to freeze small numbers of sperms.

### Polymerized alginic acid capsules

The empty capsule of a non-biological carrier is similar to empty ZP obtained from an animal. In 2006, Herrler et al. [25] developed a method of freezing small amounts of spermatozoa in polymerized alginic acid drops. Alginic acid is a chemically inert mixture of two sugars, namely, β-D-mannuronic and α-L-guluronic acid. Alginate capsules containing sperm were placed in cryoprotective agent (CPA) and frozen in straws using a programmable freezer. On thawing, alginate beads were dissolved in a sodium citrate solution. Alginate is a non-toxic polysaccharide, and polymerized alginic acid beads have been successfully used for the cryopreservation of hepatocytes [51] and stem cells [52]. The advantages of using alginate as a single-sperm freezing carrier are its gel liquid properties and its chemical inertness. Cryopreservation of human sperm by this protocol resulted in a decreased motility of about 20% compared with standard protocols. This was most likely caused by the alginic acid covering the surface of the sperm [25]. Owing to the complexity of the entire operation and other shortcomings, the developments in this technology are slow, thereby limiting its clinical application.

### Hollow-core agarose capsules

**Fig. 2:**
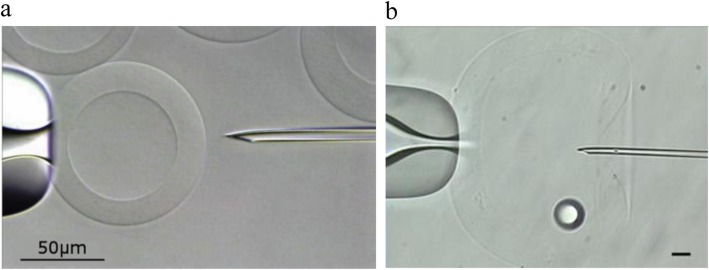
(**a**) (Adapted from Araki et al. [27]) Hollow-core agarose capsule (outer diameters, 80–120 μm; inner diameters, 60–100 μm) held by a holding pipette before injection. (**b**) (Adapted from Tomita et al. [28]) The needle was inserted into the hollow hyaluronan-phenolic hydroxyl microcapsules and one spermatozoon was slowly released. The average diameter of capsules was 241.6 ± 30.4 μm and thickness of the HA membrane was 29.6 ± 7.2 μm (*N* = 38). Scale bars = 20 μm

In the last decade, the use of empty capsules for single-sperm freezing has developed slowly. In 2007, Isaev et al. [26] used empty agarose microspheres (100-μm diameter) as a non-biological analog to ZP. One to ten spermatozoa were added into each agarose microsphere and placed in CPA solution for 5 min. One to five agarose microspheres were put into a 250 μL plastic straw. The straws were exposed to LN_2_ vapor for 10 min and then plunged into the LN_2_. After thawing, the sperm recovery rate was 98%, and 78% of recovered spermatozoa were motile. The supravital rate was 81%. In 2015, Araki et al. [27] designed hollow-core agarose capsules of size comparable with the mammalian oocytes (Fig. 2a), which were effective cryopreservation methods for individual sperm. Agarose gels had a mesh structure [53] with the advantage of being able to adjust the volume of solution on the sheet for ease-of-operation. Each agarose gel was loaded with one sperm and placed onto the mesh or polycarbonate sheets containing 0.25 to 0.5 μL cryoprotectant solution. They were frozen in LN_2_ vapor for 10–30 s and then immersed into LN_2_. Sperm resuscitation was performed in mineral oil at 37 °C. The recovery and survival rates of the sperm exceeded 90%, with activity rate of ≥80%. Spermatozoa were identified over a short time frame (10 s) after thawing, and they remained in the capsule. Agarose gel materials exhibit low toxicity, and therefore permit the culture of mammalian cells [54]. The hollow-core agarose capsules are relatively new and require further trials and investigations. More sperm function testing data will be needed to assess a potential carrier for clinical application.

### Hollow hyaluronan-phenolic hydroxyl microcapsules

In 2016, Tomita et al. [28] showed that hollow hyaluronan -phenolic hydroxyl (HA-Ph) microcapsules could be used for the cryopreservation of small numbers of sperms with no adverse effects on sperm quality (Fig. 2b). Three sperm were injected into a hollow HA microcapsule using a micromanipulator. Capsules were placed into Cryotop devices containing 1 μL of cryoprotectant. Cryotop devices were placed at 4.5 cm above the LN_2_ vapor for 2 min and plunged into LN_2_. After thawing, all frozen microcapsules were recovered, and most of the sperm were recovered in hyaluronidase-decomposing HA-Ph gels after only 2.5 min. The sperm recovery rate was 95%, which was higher than that of the empty ZP carrier. The proportion of active sperm was 14% after the resuscitation. The study showed that hollow HA-Ph microcapsules with diameter differences of several tens of micrometers do not influence sperm motility after freeze-thawing [28]. Hyaluronidase does not influence sperm motility. The recovery rates of motile sperm were relatively low owing to the freezing of the aggregated gelatin, thereby causing damage to the sperm plasma membranes [15]. Recovering spermatozoa through degradation of the capsules is favorable because it prevents physical damage to the nuclear structure.

HA-Hp microcapsules represent non-biological carriers, no ethical problems exist. However, knowledge of the clinical outcomes of this procedure is lacking, and further studies in this area are required.

### Single-sperm cryopreservation within non-biological carriers (vitrification devices)

In the past 20 years, the rapid development of the microdrop single-sperm freezing methods has led to the design and invention of various carriers. The use of suitable cryoprotectants (Table 3) can achieve better freezing effect. It is beneficial to clinical use.

**Table 3:** Summary of non-biological carriers (vitrification devices) and cryoprotectants

Non-biological carriers (Vitrification devices)	Cryoprotectants
Cryoloops	Test yolk buffer (TYB) with 12% v/v glycerol (Irvine Scientific, USA) [29]. With or without glycerol. Test-egg yolk-glycerol (TEYG) freezing medium (Scandinavian IVF Science, Gothenburg, Sweden) (glycerol concentration 12%); Standard medium (no cryoprotectant) [55]. A 50:50 mixture of HTF-HEPES with 6% plasmanate and the TYB–glycerol (Irvine Scientific, USA) cryoprotectant [30]. A 50:50 mixture of TYB–glycerol (Irvine Scientific, USA) and m-HTF medium supplemented with 6% Plasmanate [31].
Culture dish	A 50:50 mixture of IVF-20 medium (JCD, France) and sperm freezing medium (Medicult, Denmark) [33]. Sperm freezing medium (Medicult, Denmark) [34].
Cell Sleeper	A mixture of 0.7 mL SpermFreeze (FertiPro, Belguim) and 1.0 mL HFF99 (Fuso Pharmaceutical Industries, Japan) containing 20% serum substitute supplement (SSS) (Irvine Scientific, USA) [35]. SpermFreeze (FertiPro, Belgium) containing 20% SSS (Irvine Scientific, USA) [36]. A 50:50 mixture of SpermFreeze solution (Vitrolife, Goteborg, Sweden) and human serum albumin (HSA) supplemented (10 mg ml^− 1^, by manufacturer) Sydney IVF Gamete Buffer (Cook Medical) [37].
Cryotop	A mixture of 0.7 mL SpermFreeze (FertiPro, Belguim) and 1.0 mL HFF99 (Fuso Pharmaceutical Industries, Japan) containing 20% SSS (Irvine Scientific, USA) [35]. With or without glycerol. Two different cryoprotectants, sucrose (Sigma, USA) and SpermFreeze (FertiPro, Belguim) were tested. SpermFreeze includes both glycerol and sucrose. The sucrose-based freezing medium was 0.1 M sucrose in HFF99 (Fuso Pharmaceutical Industries, Japan) containing 20% SSS (Irvine Scientific, USA). SpermFreeze-based freezing medium was a mixture of SpermFreeze (0.7 mL) and HFF99 (1.0 mL) containing 20% SSS [38].
Cryoleaf	12% (v/v) glycerol and 20% (v/v) egg yolk in 0.1 M citrate buffer (PH = 7.2) [39].
Cryopiece	Freezing medium used [40].
Closed slice	With or without cryoprotectant. A 50:50 mixture of commercial sperm cryoprotectant (Quinn’s Advantage SAGE, USA) and HEPES buffer with 10% HSA. The without cryoprotectant group used HEPES buffer with 10% HSA [41]. HEPES buffer with 10% HSA [42].
The novel sperm vitrification device	A 50:50 mixture of Quinn’s Advantage Sperm Freezing Medium (SAGE, USA) and Quinn’s Sperm Washing Medium [43].

### Cryoloops

**Fig. 3:**
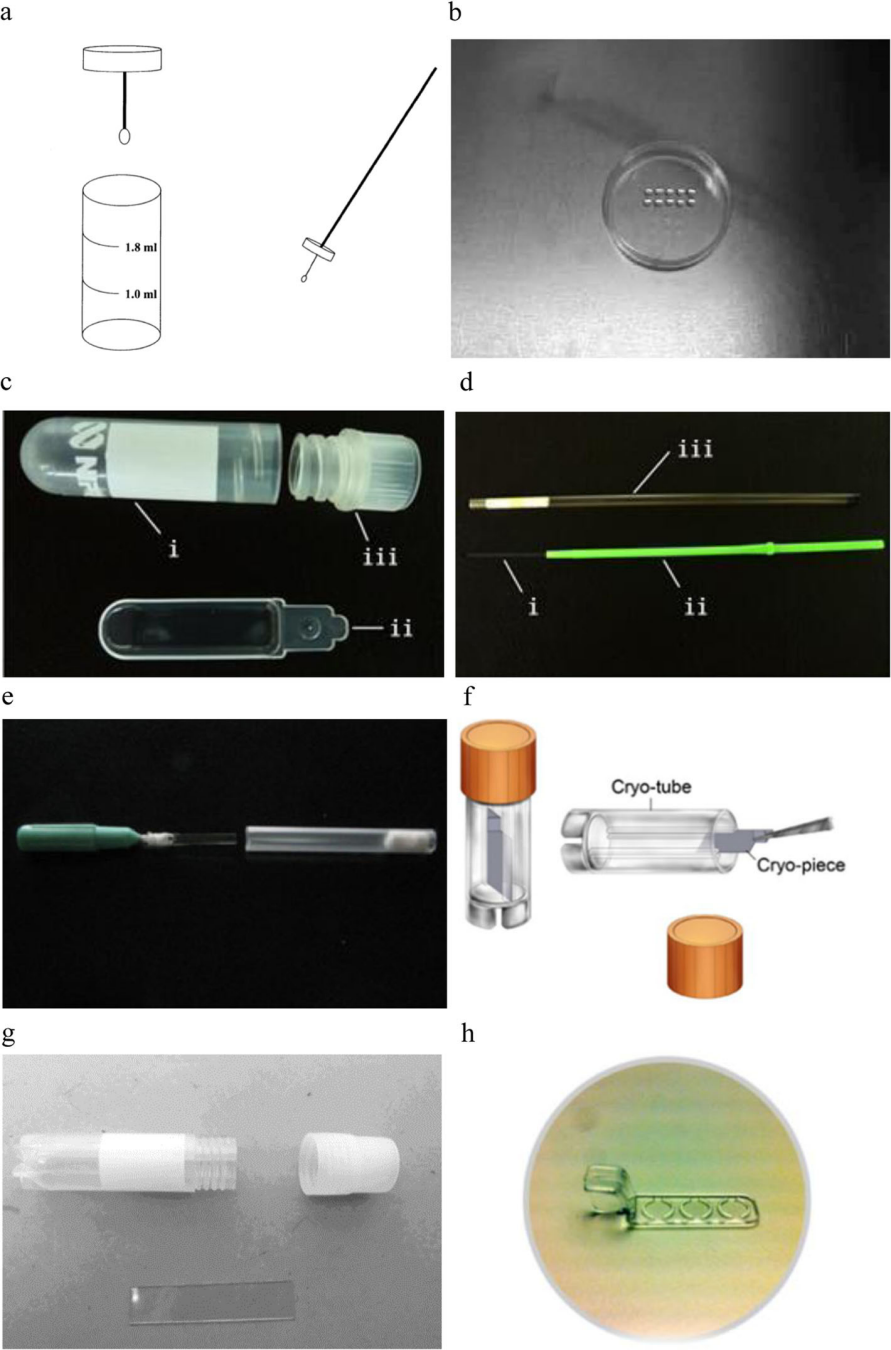
(**a**) (Adapted from Schuster et al. [29]) Cryoloops can be magnetically attached to a metal wand for easier manipulation. (**b**) (Adapted from Sereni et al. [34]) Microdrop in a plastic dish. (**c**) (Adapted from Endo et al. [35]) Cell Sleeper vial (i) is equipped with an inner tray (ii) and a screw cap (iii). (**d**) (Adapted from Endo et al. [35]) Cryotop comprises a fine polypropylene strip (i), plastic handle (ii), and cover straw (iii). (**e**) (Adapted from Peng et al. [39]) A cryoleaf bound to a handle by cotton thread is placed to a protective casing containing cotton wool at the bottom. (**f**) (Adapted from Sun et al. [40]) Droplets and polypropylene piece of cryopiece system. (**g**) (Adapted from Ma et al. [41]) The closed slice frozen carrier comprised self-made non-toxic polypropylene flakes and conventional sperm cryovials. (**h**) (Adapted from Berkovitz et al. [43]) The sperm vitrification device

Cryopreservation of individual spermatozoa is a challenging task. Owing to the shortcomings of the empty ZP method, an effective non-labor intensive methodology was developed for the cryopreservation of individual spermatozoa. Using an open cryoloop enclosed in a vial could be an alternative option (Fig. 3a). Cryoloops have been employed with success for oocyte and embryo vitrification procedures [56–59], and Schuster et al. [29] used cryoloops for the cryopreservation of small aliquots of spermatozoa. In this method, spermatozoa were submerged in experimental test yolk buffer supplemented with 12% v/v glycerol, followed by freezing in LN_2_ vapor for 5 min and snap freezing in LN_2_. Cryoloop-mediated “ultra-rapid freezing” of the oligo sperm is both simple and fast, and in this study, it resulted in 45% motility rate [29]. Moreover, successful vitrification of human spermatozoa has been achieved on cryoloops in the absence of cryoprotectants [14, 55] and this technique can be easily implemented. The post-thaw viability has been reported to be 52%, and sperm function was retained [14]. The DNA integrity and motility of the vitrified spermatozoa frozen on cryoloops were shown to be comparable with that observed for conventionally frozen spermatozoa [55]. To our knowledge, no pregnancies have been reported using vitrified/warmed sperm.

In 2004, Desai et al. [30, 31] proposed a novel cryoloop method as an alternative to hamster zona for freezing individual sperm. In this study, the authors developed a nylon cryoloop to cryopreserve 5–10 ejaculated spermatozoa, with a total of 77 spermatozoa frozen in 10 cryoloops. After thawing, the recovery and motility rates were 68% and 73%, respectively. No difference in post-thaw motility was observed after cryopreservation on loops compared with conventional vials. These individually cryopreserved sperm were indeed capable of inducing sperm head decondensation and pronuclear formation when injected into human oocytes [30]. Each nylon cryoloop was also developed to cryopreserve 2–8 epididymal or testicular spermatozoa, and they achieved 72% recovery. Sperm motility involved minimal head motion and the cryopreserved epididymal and testicular spermatozoa (including non-motile sperms) could fertilize oocytes by using conventional ICSI methods [31].

Cryoloops can be commercially purchased and they require no additional preparation. Because no animal products are required for cryoloops and the materials are non-biological, bioethical problems are avoided, making it more favorable than the hamster ZP. However, the ventilation ports of the cryovials do act as open carriers, thereby posing a risk of LN_2_ leakage and cross-contamination. Frozen droplets were carried on the surface film, thereby making the storage system unstable. So far, this method has rarely been used.

### Culture dish

Another simple method of freezing involves the replacement of the ZP by a small drop of freezing medium and its placement in a culture dish to be frozen (Fig. 3b) [32–34]. In 2000, Quintans et al. [32] cryopreserved 4–6 spermatozoa in a microdroplet with oil overlay in a plastic tissue culture dish. The dishes were sealed and stored in LN_2_, and the post-thaw recovery rate was 90–100% [32]. In 2003, Bouamama et al. [33] described single-sperm cryopreservation in culture dishes using microdroplet freezing. In this procedure, 1–100 spermatozoa were added to freezing media-microdroplet (volume: 0.5 μL) in paraffin oil, and the culture dish was sealed and stored in LN_2_. After thawing, complete (100%) recovery rate was observed and sperm movement rate was 50%. Sperm function was not further assessed. In the classical straw technique, no sperms were available following freezing-thawing of 1–10 sperms per straw. The cryopreservation of single sperm in the culture dish achieved high recovery rates compared with the classical straw technique [33]. In 2008, Sereni et al. [34] used a similar technique to freeze individual testicular spermatozoa. The total number of frozen spermatozoa was 431 (2–300 spermatozoa/sample) across six patient samples. Cryoprotectant was added to the culture dish. Before freezing, the sperm motility rate was 3.5%, and after thawing, 67% of motile sperm retained their motility. The sperm recovery rate was 100%. A total of 51 oocytes were treated across the six study samples, and in two out of six cases, motile spermatozoa were injected. The fertilization rate was 18% [34].

This method is simple and easy to control, but polystyrene culture dishes with microdroplets are problematic because their size and shape make them more difficult to store for a long term in liquid nitrogen and they cannot be sealed to create a closed system. Therefore, the risk of potential cross-contamination may increase. To date, the clinical application of the cryopreservation of individual sperm in the culture dish remains limited.

### Cell sleeper

There remains no consensus regarding the ideal carrier for the cryopreservation of individual or small quantities of spermatozoa for clinical purposes [15]. In 2012, Endo et al. [35, 36] cryopreserved small numbers of spermatozoa using the Cell Sleeper, which is a closed system. The Cell Sleeper is a vial-based cell-cryopreservation container equipped with an inner tray (Fig. 3c). Individual sperm were added to a droplet (3.5 μL) on the tray. The tray was put into a vial and sealed with a screw cap. The vial was placed in LN_2_ vapor (− 120 °C) before exposure to sterilized LN_2_. The authors applied this technique clinically in one NOA patient, resulting in the birth of a healthy boy. In addition, 12 spermatozoa from testis were vitrified. After thawing, 10 were successfully retrieved and injected individually into six mature oocytes, and the fertilization rate was 83% [35]. The effect of different vitrification volumes (0.5, 1.0, and 3.5 μL) on individually vitrified spermatozoa was measured using the Cell Sleeper. The Cell Sleeper was placed 0.5 cm above the LN_2_ vapor for 2.5 min before exposure to sterilized LN_2_. After thawing, all spermatozoa were recovered and DNA integrity was maintained. The viable sperm rate was significantly higher when spermatozoa were frozen in a 3.5 μL droplet rather than in 0.5 μL (*P* < 0.01). Frozen sperm were comparable with fresh sperm in terms of apoptotic DNA fragmentation [36]. In 2016, Coetzee et al. [37] used Cell Sleepers to cryopreserve the testicular sperm. Five to twenty-seven spermatozoa were added to a 2 μL droplet of cryopreservation solution on the tray. The Cell Sleeper was placed 4–5 cm (-115 °C– -130 °C) above the LN_2_ vapor for 2 min and then plunged into the LN_2_. A total of 304 spermatozoa were frozen in 20 Cell Sleepers, and of these, 265 were warmed. The sperm recovery rate was 94%, and the motility rate was 56%. Recovered thawed spermatozoa were injected into 179 mature oocytes, and the fertilization rate was 66% [37]. Cryopreserved immotile testicular spermatozoa can be used to inject oocytes, thereby resulting in normal fertilization [60]. Therefore, the Cell Sleeper method is fast, easy, and simple to complete. However, one drawback is the time taken to search for the sperm after thawing, which takes approximately 30 min (this period is relatively longer than that in other procedures). In addition, microsurgery requires constant lifting and lowering of the ICSI needles, thereby leading to a higher risk of needle breakage.

### Cryotop

Cryotop is a mature commercialized carrier. Cryotop, comprising fine polypropylene strips, a plastic handle, and a cover straw, has been intensely studied as a method to freeze single sperm in microdroplets (Fig. 3d). In previous studies, small numbers of spermatozoa were frozen using various types of containers. However, the lack of proper technology remains a drawback [15]. Cryotop acts as a vitrification container for both embryos and oocytes, and this system achieved 99% post-thawing survival rates [61]. Cryotop methods are also suitable for the cryopreservation of small numbers of sperm. Endo et al. [35, 36, 38] developed a simple novel vitrification technique for a single spermatozoon with the use of Cryotop at room temperature. Individual sperm were transferred to a droplet of freezing medium (1 μL) on the Cryotop strip, and the strip was immediately placed at approximately 4 cm above the surface of the LN_2_ for 2 min (− 120 °C), then directly exposed to sterilized LN_2_. Resuscitation was performed at 37 °C. One hundred spermatozoa frozen using Cryotop or empty ZP as containers had comparable rates of sperm recovery and motility after thawing. Two different cryoprotectants, SpermFreeze and sucrose, were tested, and similar rates of recovery after thawing were observed; however, the motility rate was significantly lower when sperm was frozen in SpermFreeze compared with sucrose. Sperm from ejaculates and testis were frozen using Cryotop, and the recovery and motility rates after thawing were 93% and 40%, respectively [38]. The authors applied this technique clinically in two patients with severe oligozoospermia or nonobstructive azoospermia (NOA). Eighty-one spermatozoa from testis in 8 containers (10.1 sperm per container) were vitrified, and 10 of these were warmed. All were successfully retrieved and injected individually into four mature oocytes. The fertilization rate was 75% [35]. This shows that the Cryotop method can effectively cryopreserve testicular sperm collected by micro-TESE. In another study, spermatozoa from ejaculates were cryopreserved in the Cryotop and the Cell Sleeper. After thawing, sperm recovery rate was relatively high (≥96%) in both container groups. Motility and viability of spermatozoa were similar between the two containers [36]. In 2015, Chen et al. [62] vitrified ten human spermatozoa without CPAs in 0.5 μL of freezing medium on a Cryotop strip. Both sperm recovery and motility between the sperm cryopreserved without CPAs and/or sucrose showed no significant differences. Thawed sperms without CPAs displayed higher viability and lower chromatin or DNA damage compared with samples cryopreserved with sucrose.

Cryotop is a non-biological material and does not pose any ethical issues. This technique is particularly valuable to patients with severe azoospermia and oligoazoospermia. Cryotops are now employed in ≥40 countries, and their use is documented in ≥100,000 clinical cases of oocyte and embryo cryopreservation (personal communication from Kitazato Biophrma) [38]. Healthy babies have been born with the aid of the Cryotop method.

Currently, the Cryotop vector is mainly used for freezing embryos and for freezing rare spermatozoa. In recent years, to the best of our knowledge, some new carriers, including cryoleaf, cryopiece, and closed slice, have been designed on the basis of the Cryotop method in China.

### Cryoleaf

In 2011, one study [39] demonstrated a novel method for freezing and recovery of individual or small numbers of spermatozoa. Small numbers of sperms were cryopreserved on a self-designed cryoleaf under high humidity conditions cryoprotectant that prevents evaporation (Fig. 3e). Individual spermatozoa were transferred into a droplet (0.2 μL) comprising 0.1 μL seminal plasma and 0.1 μL cryoprotectant. The cryoleaf was subsequently sheathed with a casing and then slowly lowered (20 cm in 30 s) to 1 cm over LN2 surface, exposed to the vapor for 2 min, and then plunged into the LN_2_. In this method, sperm obtained through percutaneous epididymal punctures had a motility recovery of 93% after freezing and rewarming; ejaculated sperm had a motility recovery of 62%. Following ICSI injection, no differences in the rates of fertilization were observed between fresh and frozen-thawed spermatozoa and the cleavage rates were almost similar [39]. Therefore, the cryoleaf method can effectively and conveniently cryopreserve individual sperms. However, skilled operators are required, and the freezing process must be rapid to prevent evaporation of the droplets. The technical requirements of this technique limit its widespread use in clinical practice. No successful pregnancy has been reported using this technique.

The cryoleaf vector have limited clinical use owing to their shortcomings, and the cryoleaf vector was consequently replaced by the cryopiece vector in 2016.

### Cryopiece

In previous studies [37, 38], the Cryotop and cryoleaf were developed to store the spermatozoa obtained from patients with severe oligozoospermia. Considering the potential risk of cross-contamination in LN_2_ and possible fertilization failure, a novel high-efficiency storage device and system, cryopiece technology, were developed to cryopreserve individual sperm or small sperm numbers. The cryopiece carrier (Fig. 3f) has been commercialized in China. Using this method, Sun et al. [40] from the Shanghai First People’s Hospital reported the birth of the first single-sperm cryotube baby in China in 2016. They applied this technique clinically in four patients with severe oligozoospermia or NOA. Spermatozoa with normal morphologies and higher motility were added to droplets on the cryopiece. The freezing tubes were placed into LN_2_ vapor for 15 min and subsequently plunged in LN_2_. After thawing, 83% of the spermatozoa completely recovered with movement rate of 48%. Following ICSI, fertilization rate was 73%, and 19 (86%) zygote cleavage events were observed. Four healthy babies were born at term [40].

### Closed slice

The microdrop single-sperm freezing method has been effectively employed in clinical practice. In 2015, Ma et al. [41] employed the closed slice method to rapidly freeze individual sperms in 2015. The frozen carrier comprised self-made non-toxic polypropylene flakes and conventional sperm cryovials (Fig. 3g). The effects of different vitrification volumes (0.5, 1.0, and 3.5 μL) on individually vitrified spermatozoa were compared using the closed slice method. Ten forward-moving spermatozoa were placed into droplets at 1 cm from the surface of the LN_2_ for 2 min, followed by LN_2_ plunging. After thawing, no significant differences were observed in the sperm recovery rate, motility rate, or viability rate among the three groups. Compared with techniques using frozen protective agents and those lacking cryoprotectants, the sperm recovery rate, activity rate, and survival rate were not statistically different. Closed slice vitrification methods could effectively freeze the testicular sperm in patients with obstructive azoospermia, and these methods showed a high sperm recovery and survival rate after thawing (92% and 66%). The frozen and fresh groups had comparable cleavage and fertilization rates [42].

### The novel sperm vitrification device (SpermVD)

The novel SpermVD represents an efficient carrier to freeze small numbers of spermatozoa, which optimizes fertility preservation (Fig. 3h). The SpermVD is similar to cryopiece. Sperm can be transferred to 0.8–1 μL microdroplets on the SpermVD, and 3.6 mL cryovials containing the SpermVD can be submerged into LN_2_. The time to search for spermatozoa after thawing was shortened from hours to minutes, and the sperm recovery rate reached 96%. The motility rate was 33%. The sperm were successfully used for fertilization. The fertilization and pregnancy rates were reported to be 59% and 55%, respectively. The delivery rate was 32%, and the miscarriage rate was 29% [43]. The cryopreservation of motile sperm using the novel SpermVD vector yielded improved clinical outcomes but immotile sperm were not preserved using this device, thereby limiting the knowledge regarding the efficacy of the SpermVD for immotile sperm from this source.

## Conclusions

In the last few decades, several studies have suggested a declining trend of human semen quality [63–67]. Some genetic and pathological factors are known [6], but the exact cause remains unclear. Environmental and/or occupational factors along with lifestyle practices could contribute to the deterioration of semen quality [68–71]. Lifestyle factors include cigarette smoking, alcohol intake, diet, obesity, use of illicit drugs, advanced paternal age, psychological stress, and caffeine intake. Individuals are exposed to each risk factor for varying durations and degrees of severity [72]. Awareness and recognition of the possible impact of risk factors present in daily life is crucial. In special situations, cryopreservation of sperm is also a way to avoid risks. Perfecting freezing technology will ensure better freezing effects with different qualities of semen. For example, patients with severe oligozoospermia and nonobstructive azoospermia have very limited numbers of viable sperm. Conventional cryopreservation techniques are inadequate for preserving individually selected sperm, but single-sperm freezing technology gives hope to these patients.

Since the inception of single-sperm freezing, studies on carriers and freezing methods have improved the recovery and activity rates of sperms after cryopreservation. From the use of the initial empty ZP biocarriers to the current non-biological carriers, different types of carriers have been designed for freezing small numbers of spermatozoa under different laboratory conditions. Although sperm using a single sperm freezing method by assisted reproductive technology can result in a healthy baby, to date, an ideal container that can be universally used is yet to be developed. Each cryopreservation method has its own advantages and disadvantages. As a non-biological carrier, the empty capsule freezes single sperm, promotes high sperm recovery, and high sperm movement rates. Moreover, the time to search for the recovered sperm is short. However, owing to the complexity of vector production and the lack of methods to effectively preserve the carrier, the development of the non-biological carriers is slow. Further, single-sperm microdroplet freezing methods were developed, with frozen carriers composed of polystyrene and polypropylene representing safe and simple manufacturing procedures that reduce experimental costs. In these methods, the micromanipulation process is convenient and sperm recovery rates, movement rates, and fertilization rates are high. However, this technique has not been widely used because skilled operators are required to prevent the evaporation of the droplets in clinical settings, the recovery process and the time to search for sperm are prolonged, and there is paucity of clinical data on its effectiveness. Current evidence fails to completely support the benefit of one technology over another. Every frozen sperm sample is at a risk of being contaminated. This contamination could be caused by the sample itself carrying viruses and bacteria, the non-sealed nature of the frozen carrier, and non-clean liquid nitrogen. Therefore, patients who store sperm need to be screened for the presence of HIV, hepatitis B and C, syphilis, and bacterial infections. Furthermore, the materials of the infected cases should not be stored or they should be stored in separate storage tanks, reducing the risk of virus transmission. However, the risk of contamination cannot be completely eliminated because of the possibility that patients cannot be screened for a disease. If the patient is infected, each straw may contain many viral particles. Washing the spermatozoa by a buoyant density gradient and resuspending them in a suitable sterile medium can reduce the presence of the virus below detectable levels [73]. From a safety point of view, a sealed refrigerated carrier should be used to ensure that external contamination is avoided. The laboratory must have standard operating procedures for cleaning and maintaining liquid nitrogen containers to minimize the possibility of environmental pollution [74]. Compared with ordinary sperm vitrification, single-sperm cryopreservation method uses ICSI needles to add sperm to a sterile cryoprotectant, which reduces the potential risk of virus contamination. However, due to the low rate of clinical use, there is no data on the sperm transmission contamination of the single-sperm cryopreservation method.

### Future perspectives

Because individual sperm freezing is beneficial for patients with poor sperm quality and azoospermia, early sperm optimization is of particular importance. To date, the majority of methods use traditional density gradient centrifugation to optimize sperm yields. This highlights the large developmental potential of these procedures and the need to improve the methods of single-sperm freezing technology in the future.

An array of cryoprotectants has been described. The major component of these protectants is glycerin, which reduces freezing damage and improves sperm quality. To date, however, there have been limited improvements in the use of cryoprotectants. During single-sperm droplet freezing, no cryoprotectant has achieved optimal freezing results, mainly owing to the freezing procedure and droplet volume. In-depth studies are required to establish clear reference standards.

In future research, well-designed clinical trials are required for assessing the feasibility and efficiency of various spermatozoa freezing methodologies. The goal is to provide health clinics with high-quality sperm for assisted reproduction. Further developments in this area can reduce the fertility treatment cycle, thereby improving both fertilization and pregnancy rates.

## Data Availability

Literature research results are available from the authors upon reasonable request.

## Abbreviations

CPA: Cryoprotective agent
HA: Hyaluronan
ICSI: Intracytoplasmic sperm injection
LN_2_: Liquid nitrogen
NOA: Nonobstructive azoospermia
TESE: Testicular sperm extraction
VD: Vitrification device
ZP: Zona pellucida
